# Should we consider styptics in patients with bleeding disorders?

**DOI:** 10.1016/j.rpth.2026.103403

**Published:** 2026-03-05

**Authors:** Jecko Thachil

**Affiliations:** Department of Haematology, Manchester University Hospitals, Manchester, United Kingdom

Dear Editor:

Hemostasis specialists are aware that the first response to a vessel injury is vasoconstriction. It would be ideal to have vessel contraction augmented to minimize bleeding after vascular injury. Styptics are medications that arrest hemorrhage by causing the contraction of blood vessels. The word “styptic” is defined by the Oxford English Dictionary as “a substance having the power of contracting organic tissue and works as a remedy for haemorrhage” [1]. These classic remedies have been used for centuries to stop bleeding and have recently seen a resurgence.

There is mention of styptics use since at least the 15th century, during the times when hemorrhage was the bugbear of every operating surgeon. In his “Treatises,” John Arderne recommended the excellent, simple, and clean method of sponge pressure and a styptic powder to arrest immediate hemorrhage [2]. Over the next 4 centuries, the use of styptics was mainstream to “join together” the wounds in the gut and improve bleeding diseases in women [1]. A “styptic pencil” also became commonplace among barbers and was advertised in the 1908 Sears catalog as “Used when shaving. Will instantly stop bleeding” [1]. Styptics started being utilised for more than just cuts and wounds in the First World War. The eminent botanist Bayley Balfour and military surgeon Charles Cathcart announced the official medicinal use of peat moss (sphagnum) for staunching bleeding and wound healing, although it was already widely used in Germany for this purpose [3].

Despite their widespread use, the mechanisms of action of styptics were not always clearly understood. One of the popular styptics, Monsel’s solution (ferric sulfate with sulfuric and nitric acids), was thought to achieve hemostasis by the ability of ferric iron to denature and agglutinate proteins (termed “ferrugination”) [4].

Recently, there has been a resurgence of styptics, mainly for use in the prehospital setting for trauma patients, and, interestingly, in a recent paper in the Journal, extraterrestrial regolith for use in space [5]. Regolith (the loose dust and debris from the Moon and Mars) simulants were shown to activate clotting by 3 different methods: clotting turbidity, thrombin generation, and factor XIIa chromogenic assays [5]. The researchers suggest the use of these regoliths, impregnated onto cotton or polyester gauzes and held in place, may be efficacious in stopping hemorrhage in situations where emergency surgeries, such as those following injuries to the extremities or acute abdominal complications, may result in significant bleeding [5].

Returning to the blue planet, the currently available styptic formulations are categorized under hemostats and include mechanical hemostats (eg, microfibrillar collagen) and hemostatic dressings (eg, zeolite and chitosan preparations) [6]. Some of these can work even in the absence of an intact hemostatic system and may be useful in patients with bleeding disorders. For example, zeolite acts by absorbing blood (hygroscopic action) at the site of injury and releasing calcium to initiate coagulation [7]. Chitosan renders its procoagulant function via its positively charged amino group which creates direct electrostatic interactions with the negatively charged cell membranes of erythrocytes [8].

The benefits of hemostats have been taken into consideration in international guidelines for trauma and emergency care. They suggest the use of topical hemostatic agents in combination with direct pressure to control significant hemorrhage in the prehospital setting, based on data from animal models where reduced bleeding was observed with them [9]. One of the key benefits attributed to these hemostats is the safety from thromboembolic complications occasionally noted with the systemic use of hemostatic agents [9]. Nanotechnology has also come into the fray with self-assembling peptides and nanofibers capable of stopping bleeding in a few seconds [10]. Should these useful agents, however, be limited to trauma patients or combat settings?

There are several clinical scenarios where patients with hemostatic disorders may benefit from these styptics.

• As home treatment for those who sustain cutaneous and subcutaneous injuries.
• Minimize bleed-related complications from traumatic wounds for those who live long distances from a specialist hemostasis unit.
• Hemorrhage control for external injuries in those with limited access to factor concentrates (low-resource settings).
• As an alternative for those allergic to antifibrinolytics or who have developed thrombosis (and thus cannot be prescribed antifibrinolytics).
• As an adjunct to factor concentrates in the periprocedure setting for dental and surgical interventions (this strategy may impact the duration of factor treatment).
• It is probably the only hemostatic treatment for bleeding in those with vascular disorders (however, it needs confirmatory trials).

In summary, consideration of the use of hemostats should widen from the domain of emergency caregivers and military medics to include clinicians involved in the care of patients with bleeding disorders. After all, the most famous spy, James Bond, used a styptic pencil very effectively for the cut on his chin (on the first page of the novel “Thunderball” by Ian Fleming) [1].
